# Light-dependent governance of cell shape dimensions in cyanobacteria

**DOI:** 10.3389/fmicb.2015.00514

**Published:** 2015-05-26

**Authors:** Beronda L. Montgomery

**Affiliations:** ^1^Department of Energy-Plant Research Laboratory, Michigan State University, East Lansing, MI, USA; ^2^Department of Biochemistry and Molecular Biology, Michigan State University, East Lansing, MI, USA

**Keywords:** cell division, cellular morphology, cyanobacteria, morphogenes, photomorphogenesis

## Abstract

The regulation of cellular dimension is important for the function and survival of cells. Cellular dimensions, such as size and shape, are regulated throughout the life cycle of bacteria and can be adapted in response to environmental changes to fine-tune cellular fitness. Cell size and shape are generally coordinated with cell growth and division. Cytoskeletal regulation of cell shape and cell wall biosynthesis and/or deposition occurs in a range of organisms. Photosynthetic organisms, such as cyanobacteria, particularly exhibit light-dependent regulation of morphogenes and generation of reactive oxygen species and other signals that can impact cellular dimensions. Environmental signals initiate adjustments of cellular dimensions, which may be vitally important for optimizing resource acquisition and utilization or for coupling the cellular dimensions with the regulation of subcellular organization to maintain optimal metabolism. Although the involvement of cytoskeletal components in the regulation of cell shape is widely accepted, the signaling factors that regulate cytoskeletal and other distinct components involved in cell shape control, particularly in response to changes in external light cues, remain to be fully elucidated. In this review, factors impacting the inter-coordination of growth and division, the relationship between the regulation of cellular dimensions and central carbon metabolism, and consideration of the effects of specific environment signals, primarily light, on cell dimensions in cyanobacteria will be discussed. Current knowledge about the molecular bases of the light-dependent regulation of cellular dimensions and cell shape in cyanobacteria will be highlighted.

## Introduction

Cyanobacteria and other photosynthetic organisms, which have limited mobility in their environment, are exquisitely sensitive to changes in environmental conditions. Cues that impact growth, development, and metabolism of these organisms include light, nutrients, and other factors such as inter-organismal interactions, including predation. As photosynthetic organisms depend upon light for driving energy-producing photosynthesis, these organisms are finely tuned to perceive changes in the photoenvironment. Properties of light that impact organismal form and function include light quality or prevalent wavelengths (visible and ultraviolet or UV), light intensity, daily cycles of dark/light cues, and interactions of light with other factors such as the availability of nutrients.

Light can positively promote organismal development; however, in excess it can also induce light-associated damage or phototoxicity, particularly in photosynthetic organisms (reviewed by Busch and Montgomery, 2015). Thus, changes in growth, development, or metabolism in response to light can be photomorphogenic or photoprotective in nature. Morphological changes can be linked to the optimization of the utilization of resources to support growth and development in bacterial generally (Young, 2006, 2007). A role for light in regulating cell shape or morphology occurs across many organisms, including prokaryotes (Bennett and Bogorad, 1973; Bordowitz and Montgomery, 2008; Singh and Montgomery, 2011) and eukaryotes (Daniel and Rusch, 1962; Desnos et al., 1996; Gendreau et al., 1997; Marwan, 2001; Nishihama et al., 2015). The specific mechanisms by which light impacts morphology have not been vastly studied; however, in some cases photomorphogenesis is linked to the regulation of cytoskeleton proteins (Putzer et al., 1984; Poetsch et al., 1989; Kadota and Wada, 1992; Dyachok et al., 2011; Lindeboom et al., 2013; Singh and Montgomery, 2014). Notably, the regulation of cell shape is correlated with cytoskeletal components and/or function across a range of organisms (Smith, 2003; Mathur, 2004; Cabeen and Jacobs-Wagner, 2005, 2007; Stewart, 2005; Carballido-López, 2006; Osborn and Rothfield, 2007; Terenna et al., 2008; Margolin, 2009; Ueki and Nishii, 2009).

Changes in cyanobacterial shape specifically can be directly linked to growth and/or development—light-dependent changes in vegetative cell shape (Bennett and Bogorad, 1973) or cellular differentiation, e.g., nitrogen fixation-associated development of heterocysts (Kumar et al., 2010), spore-like akinete formation for surviving harsh or stressful environments (Rippka et al., 1979; Flores and Herrero, 2010; Kaplan-Levy et al., 2010), or motility-associated hormogonium induction (Rippka et al., 1979; Meeks and Elhai, 2002; Wong and Meeks, 2002; Adams and Duggan, 2008). These developmentally induced changes in cellular morphology can be tuned by environmental cues, which include light and/or nutrient availability (Singh and Montgomery, 2011; Vadia and Levin, 2015). Stress-induced changes in cellular morphology also occur frequently in bacterial cells. UV, temperature, salt, osmotic, and biotic stresses have known impacts on the morphology of cyanobacterial cells (reviewed Singh and Montgomery, 2011). Knowledge about the mechanisms by which developmental- or stress-related cues result in morphological changes has only recently begun to emerge. Such mechanisms include light-and photoreceptor-dependent regulation of morphogene expression and cytoskeletal protein accumulation (Pattanaik and Montgomery, 2010; Singh and Montgomery, 2014), regulation of reactive oxygen species (ROS) levels that are associated with morphological determination (Singh and Montgomery, 2012; Singh et al., 2013) and light-driven changes in second messenger homeostasis that have the potential to modulate cell shape or dimensions (Neunuebel and Golden, 2008; Agostoni and Montgomery, 2014). Also noted are nutrient-associated, cell division-related cues (Goclaw-Binder et al., 2012; Osanai et al., 2013), among others.

## Light-Modulated Regulators Drive Adaptation of Cellular Morphology in Cyanobacteria

**FIGURE 1:**
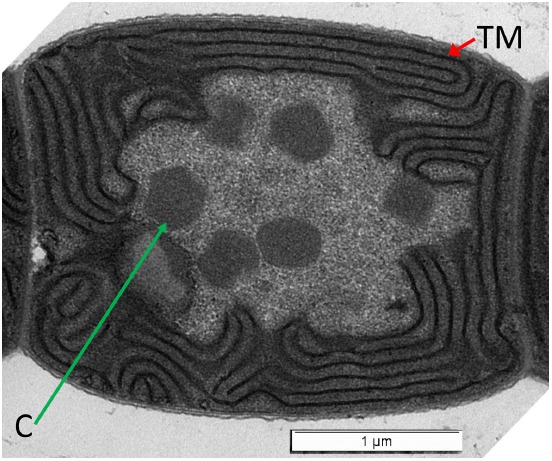
**Transmission electron micrograph of wild-type *Fremyella diplosiphon* cell grown in BG-11 medium.** Thylakoid membranes (TM; red arrow) are at the periphery of the cell. Carboxysomes (C; green arrow) are found in the center cytoplasmic region of the cell. Bar is 1 micrometer.

Light quality- or wavelength-dependent changes in physiology and development occur in some cyanobacteria (Bennett and Bogorad, 1973; Bordowitz and Montgomery, 2008). The well-studied, freshwater filamentous cyanobacerium *Fremyella diplosiphon* exhibits an acclimatory phenomenon, which is historically known as complementary chromatic adaptation or CCA, during which the photosynthetic pigment content is tuned to the external environment (Bennett and Bogorad, 1973; Tandeau de Marsac, 1991). The organism accumulates red-colored, green wavelength-absorbing pigment phycoerythrin (PE) in green-enriched environments, and conversely blue-green-colored, red wavelength-absorbing pigment phycocyanin (PC) in red-enriched environments (Gutu and Kehoe, 2012). The PE and PC pigments are contained in peripheral light-harvesting complexes called phycobilisomes (PBS). The PBS antennae are associated with the thylakoid membranes generally found at the periphery of cyanobacterial cells (Figure 1). As the availability of red vs. green wavelengths vary significantly at difference depths in a water column, CCA-dependent tuning of photosynthetic pigmentation is linked to environmental adaptation and tuning of photosynthetic efficiency in natural contexts (Campbell, 1996; Postius et al., 2001). This process is regulated by a soluble, light-absorbing photoreceptor known as RcaE in *F. diplosiphon* (Kehoe and Grossman, 1996). As a part of CCA, there are noted changes in cellular morphology, in addition to pigmentation. Spherical cells and shorter filaments are characteristic of growth under red light (RL), whereas rectangular cells and long filaments are associated with growth under green light (GL; Bennett and Bogorad, 1973; Bordowitz and Montgomery, 2008). RcaE is the regulator for cell shape and filament morphology changes, in addition to its previously mentioned role in fine-tuning photosynthetic pigment levels in *F. diplosiphon* (Bordowitz and Montgomery, 2008). RcaE mutant cells exhibit a rounded cell shape independent of the external light conditions, in contrast to the light-regulated switch between spherical- and rod-shaped cells observed for wild-type (Bennett and Bogorad, 1973; Bordowitz and Montgomery, 2008).

Apart from RcaE which is linked to the regulation of pigmentation and morphology in *F. diplosiphon*, another regulator cpeR has also been linked to the regulation of both pigmentation and morphology in this organism (Pattanaik et al., 2011b). CpeR induces synthesis of the photosynthetic pigment PE in GL (Cobley et al., 2002; Seib and Kehoe, 2002; Alvey et al., 2003). However, it was also shown that a Δ*cpeR* mutant in *F. diplosiphon* exhibits an altered cellular morphology compared to wild-type cells (Pattanaik et al., 2011b). Thus, the CpeR regulator, which appears to serve as a transcriptional activator for PE genes (Cobley et al., 2002), also likely controls genes important for photoregulation of cellular morphology. Notably, many other pigment mutants isolated in *F. diplosiphon* impact pigmentation of cells with no measurable impact on cellular morphology (Whitaker et al., 2009, 2011; Bordowitz et al., 2010; Pattanaik et al., 2011a,b).

Light intensity has been shown to impact cellular morphology in cyanobacteria (Bittencourt-Oliveira et al., 2012; Pattanaik et al., 2012; Walters et al., 2013). *Cylindrospermopsis raciborskii* exhibits trichome elongation under reduced light intensity (Bittencourt-Oliveira et al., 2012). In *F. diplosiphon*, lower intensity light results in elongated cells, whereas light of increased intensity results in cells which are more spherical and with reduced length (Pattanaik et al., 2012; Walters et al., 2013). In natural environmental contexts, elongated *F. diplosiphon* cells are prevalent in low light intensities, whereas spherical cells exist in higher intensity regions of the water column (Montgomery, 2008). Thus, it was proposed that cells may regulate cellular morphology in natural contexts under different light intensities to control cellular volume and associated capacity for total photosynthetic membrane content that is correlated with regulation of cellular photosynthetic capacity (Montgomery, 2008; Pattanaik et al., 2012; Walters et al., 2013). As related photoreceptors serve as potent light quality and intensity sensors (e.g., Reed et al., 1994; Rausenberger et al., 2010), it is proposed that RcaE serves as a mediator of light intensity-dependent regulation of morphology in *F. diplosiphon*.

Visible and UV light exposure can result in trichome spiral compression in *Arthrospira platensis* (Wu et al., 2005; Ma and Gao, 2009). The mechanism of this morphological regulation has been implicated as a periplasmic protein of undetermined function (Ma and Gao, 2009). Spiral compression in this organism may serve a role in self-shading that can protect cells against damaging UV exposure (Wu et al., 2005). Thus, the smaller, compressed state under UV has been correlated with reduced surface area that has been hypothesized to serve as a photoprotective state associated with the maintenance of photosynthetic capacity (Singh and Montgomery, 2011). Relatedly, trichome breakage or filament fragmentation under UV light also occurs in *A. platensis* and other cyanobacteria (Gao et al., 2007; Ma and Gao, 2010; Rastogi et al., 2010, 2014). Similar to trichome compression, these responses may be associated with reduced surface area potentially exposed to UV and a potential reduction in UV-associated damage.

## Light-Dependent Regulation of Morphogenes is One Mechanism for Regulating Cellular Morphology in Cyanobacteria

Although distinct links between environmental signals and modulation of cellular morphology are clear in cyanobacteria (Singh and Montgomery, 2011), knowledge about the mechanisms by which these changes are mediated is limited. TonB is important for regulating GL-dependent cellular morphology in *F. diplosiphon* (Pattanaik and Montgomery, 2010). Although its exact biochemical function has not been elucidated, the protein has sequence similarity to both iron-associated TonB proteins and a glycine-rich region related to domains in proteins involved in cellular elongation (Sachetto-Martins et al., 2000; Pattanaik and Montgomery, 2010). However, the GL-associated disruption in photoregulation of morphology in a Δ*tonB* mutant in *F. diplosiphon* was shown to be independent of responses to iron limitation (Pattanaik and Montgomery, 2010). This observation, thus, suggests a more direct role for TonB in modulating cell shape in response to GL in this organism.

Recently, photoregulation of morphogenes has emerged as a key mechanism for tuning cellular morphology to light cues in cyanobacteria (Singh and Montgomery, 2014). Morphogene function in the regulation of cellular morphology generally is well known. The bacterial actin MreB is associated with rod-shaped bacteria, and is nearly always absent in spherical bacterial cells (reviewed by Cabeen and Jacobs-Wagner, 2007). MreB has functional homologs in cyanobacteria, both filamentous (Hu et al., 2007; González et al., 2010; Singh and Montgomery, 2014) and unicellular (Koksharova et al., 2007; Savage et al., 2010; Singh and Montgomery, unpublished data), which are associated with rod-shaped cells. Mutation of *mreB* in cyanobacterial systems leads to a loss of shape regulation, thus resulting in the adoption of spherical cells in Δ*mreB* mutants (Hu et al., 2007; Savage et al., 2010). In *F. diplosiphon*, in which rod-shaped cells are prevalent under GL, it appears that photoregulation of *mreB* expression corresponds to the induction of rod-shaped morphology (Singh and Montgomery, 2014).

A well-characterized regulator of MreB is the morphogene BolA (Aldea et al., 1989; Santos et al., 1999). Accumulation of BolA is associated with a spherical cell shape, and imparts this morphology in part through transcriptional downregulation of *mreB* (Aldea et al., 1988, 1989; Santos et al., 1999; Freire et al., 2009). In the first reported functional characterization of a cyanobacterial BolA protein, we recently showed that photoregulation of *bolA* expression in *F. diplosiphon* is correlated with RL-dependent BolA accumulation that is associated with spherical morphology (Singh and Montgomery, 2014). Similar to *E. coli* BolA (Freire et al., 2009), BolA from *F. diplosiphon* binds the promotor of *mreB* to maintain low levels of *mreB* expression in RL (Singh and Montgomery, 2014). Lower levels of BolA are found in GL which are correlated with derepression of *mreB* expression, thereby resulting in the induction of a rod-shaped morphology in *F. diplosiphon* under these conditions (Singh and Montgomery, 2014).

## Additional Signals that Impact Cyanobacterial Morphology or Cellular Dimensions—ROS, Second Messengers, and Cell Cycle Factors

Additional factors apart from morphogenes have been correlated with the regulation of cellular morphology in response to environmental cues in cyanobacteria. Some factors induced by environmental cues, including light, that impact cellular dimensions include reactive oxygen species (ROS), second messengers, and cell cycle or division cues.

### ROS as a Signal that Impacts Cellular Morphology of Cyanobacteria

Reactive oxygen species are potent cues that can arise from photooxidative stress. When light absorption by the photosynthetic light-harvesting complexes exceeds the need for carbon fixation or the capacity of the photosynthetic electron transfer chains, absorbed light energy can be transferred to other targets resulting in production of ROS (reviewed by Busch and Montgomery, 2015). Generated ROS can then result in cellular photodamage and/or ROS molecules can serve as signals that impact cellular function or development (Busch and Montgomery, 2015). In cyanobacteria, light-generated ROS have been associated with the adoption of particular cellular morphologies. In *F. diplosiphon*, ROS levels are elevated in RL and this light-induced ROS accumulation is associated with spherical morphology (Singh and Montgomery, 2012; Singh et al., 2013). ROS levels are lower in GL and associated with rod-shaped *F. diplosiphon* cells (Singh and Montgomery, 2012; Singh et al., 2013). ROS specifically appear to be the signal impacting morphological adaptation as treatment of cells grown in RL with ROS-scavenging antioxidants reverses the ROS-associated impacts on cellular dimensions (Singh and Montgomery, 2012). In addition to the impact of ROS on cellular morphology, filament length is regulated by photoregulation of ROS. RL-associated elevated ROS accumulation is correlated with shorter filaments, presumably due to filament fragmentation (Singh and Montgomery, 2012). Notably, the RL vs. GL effects on ROS levels are regulated by the RL-responsive RcaE photoreceptor (Singh and Montgomery, 2012), a photoreceptor which regulates pigmentation and the photoregulation of morphology as described above (Kehoe and Grossman, 1996; Bordowitz and Montgomery, 2008). ROS levels are also elevated when light intensity is increased, which also is associated with the induction of a spherical cellular morphology (Walters et al., 2013). ROS have been associated with the regulation of cellular morphology in other systems, including non-photosynthetic organisms such as *Aspergillus nidulans* (Semighini and Harris, 2008) and specific phenotypes such as pollen germination and development, root elongation and cell expansion in plants (Foreman et al., 2003; Carol and Dolan, 2006; Li et al., 2007; Potocký et al., 2007; Müller et al., 2009; Speranza et al., 2012). One function of ROS in this regard is in cell wall loosening (Müller et al., 2009), potentially due to lipid peroxidation (He and Häder, 2002). This ROS-dependent impact could be linked to observed changes in cellular morphology.

The role of UV irradiation in spiral breakage or filament fragmentation in cyanobacteria has been associated with ROS formation (Ma and Gao, 2010; Rastogi et al., 2010, 2014). The accumulation of ROS has been implicated in the induction of lipid oxidation, which ultimately is hypothesized to lead to cellular damage and/or lysis (Ma and Gao, 2010; Rastogi et al., 2010). Thus, as in other systems, ROS molecules have the potential to serve as signaling molecules as proposed for the regulation of cellular morphology in *F. diplosiphon* or as molecules that cause potential damage such as cellular lysis (Busch and Montgomery, 2015). Ultimately, however, the morphology changes and/or filament fragmentation may be associated with improved fitness of strains in some cases. Early experiments with cyanobacteria demonstrated that targeted cell lysis at specialized cells along a filament can result in fragmentation or breakage of filaments into shorter filaments (Lamont, 1969; Bennett and Bogorad, 1973). Such filament fragmentation due to targeted cell lysis during the transition of *F. diplosiphon* cultures to RL was proposed to facilitate a reduction in the content of phycobiliproteins in the culture that are not optimal for growth in RL (Bennett and Bogorad, 1973). This morphological adaptation, thus, is associated with temporal tuning of the pigment content of the organism to the external light environment, which has been associated with optimizing photosynthetic efficiency (Campbell, 1996). Additionally, filament length regulation has been associated with fitness implications in a heterocyst-forming cyanobacterium, whereby the ability to restrict filament length under nitrogen-fixing conditions is associated with improved survival of *Anabaena* sp. strain PCC 7120 (Merino-Puerto et al., 2013).

### Second Messengers are Correlated with the Regulation of Cyanobacterial Cell Shape

Second messengers serve as key signaling molecules used in cellular responses to external (or first messenger) cues. These molecules can be rapidly turned over in an energetically conservative manner to facilitate rapid changes in response to environmental fluctuations. Cyanobacteria contain a wide range of second messengers (Agostoni and Montgomery, 2014). Calcium is a ubiquitous second messenger utilized in response to a number of environmental cues that has been implicated in several cyanobacterial responses, including heterocyst development and homorgonia differentiation (reviewed by Agostoni and Montgomery, 2014). The second messengers guanosine pentaphosphate or tetraphosphate [(p)ppGpp] and cyclic AMP (cAMP) have been implicated specifically as mediators of cellular differentiation during cellular responses to nutrient availability (reviewed by Agostoni and Montgomery, 2014). Cyclic dimeric guanosine 3′,5′-monophosphate, i.e., c-di-GMP, presumably contributes to cell shape maintenance and cellular development in the filamentous cyanobacterium *Anaebaena* sp. PCC 7120 as deletion of putative c-di-GMP synthesis enzyme-encoding gene *all2874* results in reduced heterocyst development and smaller vegetative cells under high light intensity (Neunuebel and Golden, 2008). Notably, cAMP (Ohmori et al., 1988, 2002; Ohmori and Okamoto, 2004; Okamoto et al., 2004; Terauchi and Ohmori, 2004) and c-di-GMP (Agostoni et al., 2013) levels are controlled by light in cyanobacteria. As many of these molecules have only recently begun to be the focus of studies in cyanobacteria (Agostoni and Montgomery, 2014), many additional second messenger-regulated responses are expected to emerge.

### Coordination of Cell Cycle, Cell Division, and Central Carbon Metabolism with Cell Dimensions

Cellular dimensions are largely heritable across a range of organisms, including prokaryotes and eukaryotes (Zaritsky, 1975; Fantes, 1977; Koch, 1996; Chien et al., 2012; Marshall et al., 2012; Turner et al., 2012; Lloyd, 2013; Campos et al., 2014). Cell size control is a core feature of the bacterial cell cycle under defined growth conditions (Campos et al., 2014). Bacterial cell size homeostasis appears to be regulated by reproducible and constant cell size extension (Campos et al., 2014; Soifer et al., 2014; Taheri-Araghi et al., 2015). Coordination of cell growth or elongation and division during the cell cycle maintains and/or regulates cell size in bacteria, and can be impacted by nutrient availability or central metabolism (Weart et al., 2007; Siegal-Gaskins and Crosson, 2008; Chien et al., 2012; Yao et al., 2012; Hill et al., 2013; Robert et al., 2014).

Cell division and elongation in cyanobacteria have also been correlated (Koksharova and Wolk, 2002; Mazouni et al., 2004; Miyagishima et al., 2005; Marbouty et al., 2009; Gorelova et al., 2013). Although disruptions in cell division can result in elongated or filamentous cells, in some cases, the reversion of the impairment in cell division results in the restoration of the original cellular dimensions, providing evidence for the recognized strong regulation over cell size (Goclaw-Binder et al., 2012). Environmental cues such as nutrient availability can impact cell division and thereby impact cellular morphology in cyanobacteria (Goclaw-Binder et al., 2012), as described above for other bacterial systems.

An impact of central carbon metabolism on the cell cycle can affect cell size (reviewed by Vadia and Levin, 2015). While definitive experiments linking central carbon metabolism with cell size in cyanobacteria lag behind assessment in other systems, interesting phenomena have been reported. Cell division regulation that is associated with alterations in cell size has been shown to be impacted by sugar metabolism in *Synechocystis* (Osanai et al., 2013). This observed phenotype is driven by overexpression of a sigma factor gene, i.e., *sigE*, which impacts gene expression through interacting with the RNA polymerase (Osanai et al., 2013).

## Carbon-Concentrating Mechanism and Correlations with Cell Shape

In cyanobacteria, carboxysomes are subcellular microcompartments centrally located in the cytoplasm of cells (Figure 1) and which are associated with the carbon-concentrating mechanism (CCM) and carbon fixation in cyanobacteria (Rae et al., 2013). In some studies, changes in carboxysome quantity and carboxysomal structural defects are apparent in elongated cell division mutants lacking *ftn2* and *ftn6* (Gorelova et al., 2013). Additionally, a disruption of apposite spatial distribution of carboxysomes in a mutant with cytoskeleton-associated defects in cellular morphology has been observed (Savage et al., 2010). Such observations suggest interactions between cellular shape and/or division with the spatial organization of carboxysomes central to carbon metabolism.

The CCM can also include alterations to inorganic carbon (Ci) uptake or active transport. Low Ci levels result in a reduced trichome size in *Arthrospira platensis* (Ma and Gao, 2014). This reduction of trichome size is hypothesized to increase the surface area to cellular volume ratio. Such a response may serve in part to sustain photochemical efficiency and reduce potential damage under high light conditions, which are associated with an increased CCM (Beardall, 1991; Qiu and Liu, 2004).

## Conclusion

Cyanobacteria and other cells can alter cellular dimensions and/or shape in response to environmental cues. In some cases this morphological adaptation is linked to the production of cells tuned to the external environment to increase fitness. In other cases, the fitness implications of the energy-requiring morphological adaptations are less clear or only beginning to emerge. For example, *F. diplosiphon* can alter cellular morphology in response to wavelength- or intensity-dependent cues that appear to be correlated with regulating cellular capacity for photosynthetic protein accumulation, which is linked to tuning the capacity of photosynthetic efficiency and associated carbon metabolism to external cues. Other cyanobacteria can alter trichome spiral length and/or filament morphology to reduce surface area exposed to potential damaging light, such as UV or high intensity light. Cells such as *A. platensis* and *C. raciborskii* can alter trichome size in response to cues such as light intensity and Ci availability, presumably to limit damage and alter surface to volume ratios that may alter resource acquisition to sustain photosynthesis. Additional analyses of the mechanisms by which morphological changes are regulated and the potential fitness costs and/or benefits may result in targets for engineering cyanobacterial strains for use in biotechnological applications, in addition to providing greater insight into the roles of these adaptations in natural contexts.

### Conflict of Interest Statement

The author declares that the research was conducted in the absence of any commercial or financial relationships that could be construed as a potential conflict of interest.
